# SRAP analysis of the genetic diversity of wild castor (*Ricinus communis* L.) in South China

**DOI:** 10.1371/journal.pone.0219667

**Published:** 2019-07-11

**Authors:** Kwadwo Gyapong Agyenim-Boateng, Jiannong Lu, Yuzhen Shi, Dan Zhang, Xuegui Yin

**Affiliations:** College of Agricultural Sciences, Guangdong Ocean University, Zhanjiang, Guangdong, China; National Cheng Kung University, TAIWAN

## Abstract

Castor bean is an important seed oil crop. Castor oil is a highly demanded oil for several industrial uses. Currently, castor bean varieties suffer from low productivity and high risk of insect pests and diseases. It is in urgent need to mine elite genes from wild materials for castor breeding. 29 pairs of polymorphic SRAP primers out of 361 pairs were used to analyse the genetic diversity of 473 wild castor materials from South China. 203 bands were amplified by the 29 pairs of primers, of which 169 bands were polymorphic, with a polymorphic percentage of 83.25%. With an average number of alleles per locus (A_p_) of 1.801, average number of effective alleles per locus (*A*_*e*_) of 1.713 and average percentage of polymorphic loci (P) of 90.04%, these primers were proven to be useful and effective. Nei’ genetic distance between the materials ranged from 1.04 to 25.02, with an average of 13.03. At the genetic distance of 25.02, the materials clustered into two major groups, consistent with the result of population structure analysis. However, more subgroups existed between 5.21 and 13.32. Although not all the materials from the same region were clustered in the same group, an obvious trend existed where the groups were related to regions to a great extent. Based on multiple indices, the genetic diversity of materials from Hainan was the lowest. However, there was not much difference between West Guangdong and Guangxi, although the former was slightly higher. Moderate genetic differentiation was observed in wild materials in South China. The genetic differentiation mainly occurred within population, with maximum differentiation in Guangxi, followed by West Guangdong and the minimum in Hainan. Nonetheless, there was an extensive geneflow between populations. The above results provided a direction for the conservation and breeding application of these materials.

## Introduction

Castor (*Ricinus communis* L.; 2n = 20) [1] belongs to the family Euphorbiaceae and has been cultivated even before 4,000 B.C. as shown by buried castor seeds in Egyptian tombs [2, 3]. It is said to have originated from tropical Africa precisely, Ethiopia, since there is a higher diversity of wild and semi-cultivated types of castor over there compared to any other part of the world. Castor was taken to India and China, and it now can grow anywhere in the world (non-frost areas) [2, 4] partially due to its stable rooting system and drought resistance.

Castor, which is a non-edible oilseed crop is grown extensively in the tropical, subtropical and temperate regions of the world for its highly valued oil (Castor oil). The oil from castor is used as a raw material for many industrial products owing to its unique physical and chemical properties. Castor oil has numerous industrial and medicinal applications [5] like being used as high quality lubricants for aircrafts, alternative to sulfur based polluting lubricants/additives in petroleum diesel, paints, coatings, polishes, textile dyes, surfactants, resins, plastics, waxes, soaps, medications, cosmetics, perfumes, etc. [6]. In medicinal applications, castor oil has been used since ancient times as a favourite stimulant laxative and oil inducer for pregnant women to begin labour [7], as eye-drops for lipid-deficient dry eyes [8], and as an ingredient for reducing edema, scabbing of patient wounds, and treating skin diseases [9]. Castor oil can also be used for biodiesel production as feedstock although it is more valued in the chemical industries than the petroleum industry [10, 11]. The higher presence of ricinoleic acid (80~85%), a fatty acid in castor oil, represents its uniqueness [12]. Aside from the unique chemical qualities, castor oil is the only vegetable oil soluble in alcohol at room temperature, high in viscosity and requires less heating in the production of biodiesel compared to other oils. Hence it becomes the best choice for the biodiesel preparation [13]. Castor cake, which is the by-product of castor oil extraction, is also used as manure and the fresh leaves are used to feed eri silkworm, and dry leaves are used as insecticides [6]. The castor plant has been seen as very beneficial in eri silk production. Castor plays an essential role in the economy of arid and semi-arid regions of the world because of its ability to grow under dryland conditions [14].

Genetic diversity describes the total number of characteristics in the genetic makeup of a species and provides a way for populations to adapt to fluctuating environments. For a breeding program to be effective, good knowledge of the extent and nature of genetic diversity within the crops is a necessity. The presence of genetically different landraces of a crop is a handy resource that can be used in the development of such a crop. Analysis of population structure and genetic diversity of germplasm is a very productive tool in providing information for association mapping, allele mining for novel traits and crop breeding [15].

For the possibility of proper improvement of new cultivars with required characters that are suited to specific microclimates, there should be some knowledge about the extent of genetic diversity of the species [16].

Many molecular markers like SSR (Simple Sequence Repeats), SNP (Single Nucleotide Polymorphism), EST-SSR (Expressed Sequence Tag-Simple Sequence Repeat), AFLP (Amplified Fragment Length Polymorphism), SCOT (Start Codon Targeted Polymorphism), RAPD (Random Amplified Polymorphic DNA), ISSR (Inter-Simple Sequence Repeat) and TRAP ((Target Region Amplification Polymorphism) have been used in assessing the genetic diversity of castor [17–26]. Nonetheless, irrespective of the marker systems used, the global analysis of the genetic diversity of castor germplasm shows the low levels of variability and a lack of geographically structured genetic population [17–19, 23].

SRAP (Sequence Related Amplified Polymorphism) markers since their introduction [27] have never been used in accessing the genetic diversity of castor. However, SRAP markers have been used in accessing the genetic diversity of many crops, even that of pigeon pea and cumin [28, 29]. The SRAP markers demonstrated high utility in detecting genetic variation among pigeon pea genotypes in comparison with SSR and AFLP-RGA systems and were proven to be a valuable tool in determining genetic diversity in cumin and its improvement.

The procedure of SRAP primer design is relatively easy and fast since no prior sequence information is needed and the forward and reverse primer combinations are not limited, yielding more polymorphisms. Compared with other molecular marker methods, primers designed for SRAP markers are more reproducible, stable and less complicated [27] and have been found to be more useful for revealing genetic and species diversity in closely related cultivars [30]. SRAP markers are especially suitable for the species without SSR markers as they require a comparatively little sequence information for marker development and can be used as universal primers between species. The SRAP procedure can be performed with a reasonable throughput rate and has been proved as time efficient and cheaper marker system for displaying genetic differentiation on inter and intraspecies [27, 31]. SRAP is an adequate molecular marker system for genetic diversity analysis in plants.

Castor has been planted in China over 1,400 years, and it is mainly planted in northern China. Most popularised varieties were developed in the north but expressed premature senility, weak resistance to disease and poor tolerance to heat and moist in South China, while the abundant wild and semi-wild (domesticated ancestors but found in the wild) castor plants in South China lack effective improvement and utilisation. As a result, South China lacks adaptable varieties. The objective of this study is to access the genetic diversity of castor accessions from South China using SRAP markers to provide a basis for efficient protection, research, and utilisation of these resources.

## Materials and methods

### Plant materials

**Fig 1:**
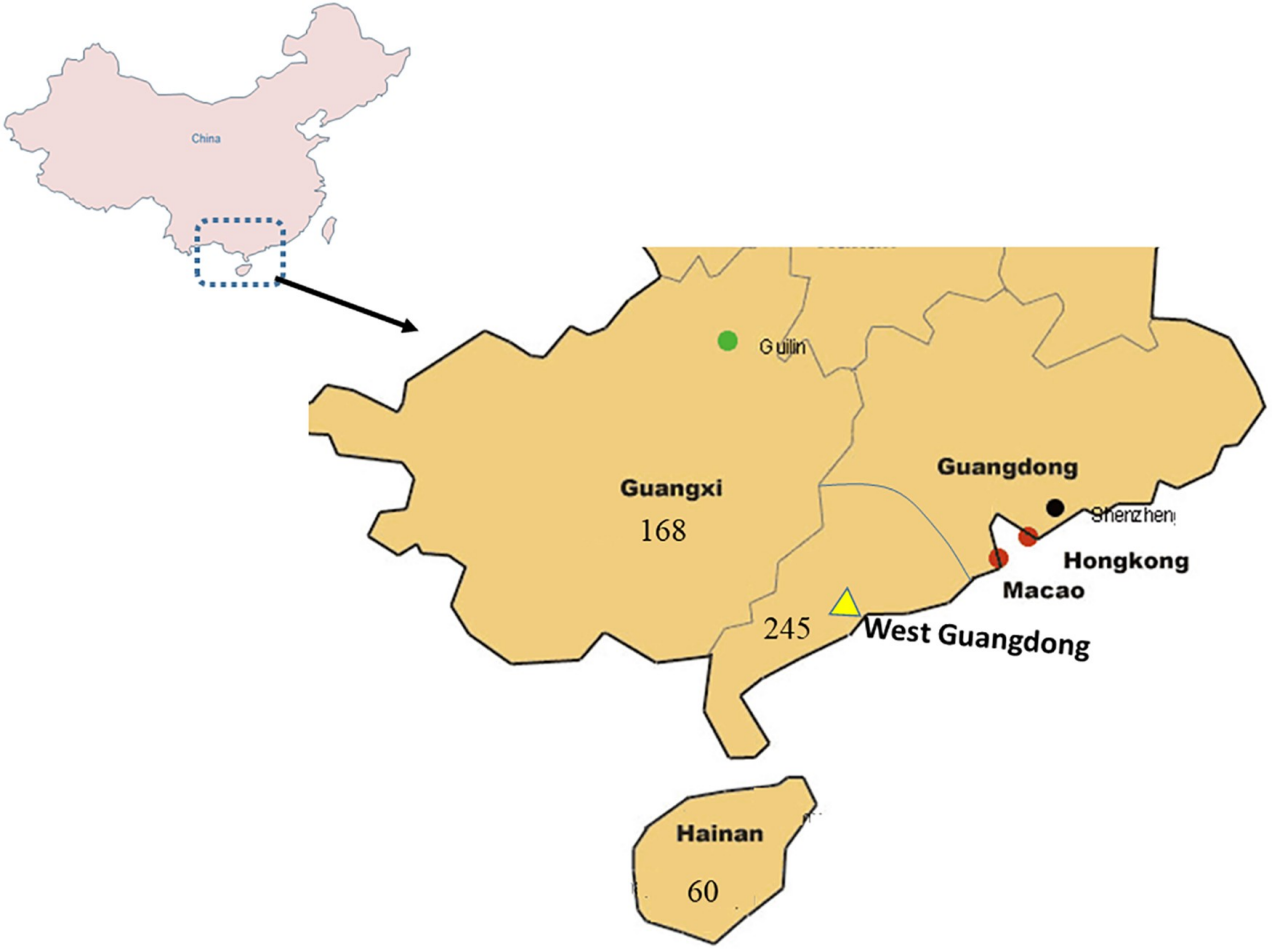
Locations of the accessions on the map of China.

All the castor materials were provided by the tropical crop molecular breeding laboratory of Guangdong Ocean University. The sampling plan was formulated as follows: On the map, the collection area was divided into stripes 50–100 kilometers apart in the north-south direction, and several roads each going across a stripe were selected; we drove along these roads, searching and collecting; at the same time, we visited local people to look for the places where castor plants were distributed. At each collection point, we collected a few to a dozen plants, and 3–5 seeds were collected from each plant. The distance between the collection points was ~50 kilometers. Actually, the castor plants at each collection point were well isolated in space. After screening and sorting, 473 materials (245 samples from West Guangdong, 168 from Guangxi and 60 from Hainan) (S2 Table) were selected from 1682 materials collected from West of Guangdong Province, Guangxi Zhuang autonomous region and Hainan Province. Generally, phenotypic characteristics such as plant height, stem thickness, number of capsules and capsule characters, chlorophyll content, wax bloom and leaf characters were observed to be quantitative in all three (3) regions. Averagely, the Guangxi region was characterized with tallest plants, highest wax bloom and number of capsules. West Guangdong, despite having the averagely shortest plants had the thickest stems and the highest chlorophyll content. Hainan on the other hand, although having moderately tall plants had the least number of capsules, chlorophyll content, stem thickness and the lowest wax bloom. Fig 1 shows the locations on the map of China where the accessions were collected. A total of 23 samples, 10, 8, and 5 were randomly selected from West Guangdong, Guangxi, and Hainan respectively to select polymorphic primers.

### SRAP materials

361 pairs of SRAP primers were prepared by randomly combining 19 forward primers, Me1-Me19, and 19 reverse primers, Em1-Em19. The SRAP primers were synthesised by Shanghai Bioengineering Company. A total of 29 pairs of polymorphic primers were selected to be scanned on the entire materials after being screened on the 23 selected materials. The 29 combinations were M1E3, M1E9, M2E2, M2E4, M2E5, M2E6, M2E7, M2E13, M3E7, M3E9, M4E1, M5E7, M6E4, M6E5, M6E6, M9E1, M9E8, M10E16, M10E17, M15E5, M15E12, M17E2, M17E3, M17E9, M17E11, M17E12, M18E10, M18E16, AND M19E1. The primer sequence is listed in Table 1.

**Table 1:** SRAP primer sequences.

Forward primers	Primer Sequence	Reverse primers	Primer Sequence
Me1	TGA GTC CAA ACC GGA TA	Em2	GAC TGC GTA CGA ATT TGC
Me2	TGA GTC CAA ACC GGA GC	Em3	GAC TGC GTA CGA ATT GAC
Me3	TGA GTC CAA ACC GGA AT	Em4	GAC TGC GTA CGA ATT TGA
Me4	TGA GTC CAA ACC GGA CC	Em5	GAC TGC GTA CGA ATT AAC
Me5	TGA GTC CAA ACC GGA AG	Em6	GAC TGC GTA CGA ATT GCA
Me6	TGA GTC CAA ACC GGA CA	Em7	GAC TGC GTA CGA ATT CAA
Me9	TGA GTC CAA ACC GGA GG	Em8	GAC TGC GTA CGA ATT CAG
Me10	TGA GTC CAA ACC GGA AA	Em9	GAC TGC GTA CGA ATT TAG
Me15	TGA GTC CAA ACC GGC AT	Em10	GAC TGC GTA CGA ATT CAT
Me17	TGA GTC CAA ACC GGT AA	Em11	GAC TGC GTA CGA ATT CTA
M18	TGA GTC CAA ACC GGT CC	Em12	GAC TGC GTA CGA ATT CTC
M19	TGA GTC CAA ACC GGT GC	Em16	GAC TGC GTA CGA ATT GCT
Em1	GAC TGC GTA CGA ATT AAT	Em17	GAC TGC GTA CGA ATT CGA

### DNA extraction

Total genomic DNA was extracted from the young leaves following the improved cetyltrimethylammonium bromide method (CTAB).). The DNA extraction method is as follows:

1. Fresh castor leaves were ground into powder using liquid nitrogen, and the grounded powder was filled to about 1/3 volume of 1.5ml Eppendorf tube;
2. 800μL CTAB buffer (pH 8.0 Tris-HCl 100mmol L^-1^, pH 8.0 EDTA 50mmol L^-1^, 3% CTAB, NaCl 100 mmol L^-1^, 4% PVP, 2% β-mercaptoethanol) was added and to grounded leaf powder and thoroughly mixed; tubes were placed in a water bath at 65 °C for 20–30 min and were shaken at every 5–10 minute interval;
3. Tubes were centrifuged at 10,000 rpm for 5 minutes at room temperature;
4. The supernatant was taken, and 6μL of RnaseA enzyme (5mgmL^-1^) was added. Incubation was done at 37 ° C for 1 hour;
5. An equal volume of chloroform: isoamyl alcohol (24:1) was added, and the solution was gently shaken to milky white (about 5 min) and was centrifuged at 10,000 rpm for 10 minutes at room temperature;
6. The supernatant was taken, and 20μL NaAc (3molL^-1^ pH 5.2) and 120μL absolute ethanol (pre-cooled at -20 °C) was added, gently mixed, and left at room temperature for 2 min. It was centrifuged at 10,000rpm for 5 minutes. The solution was poured away leaving the DNA pellet settled at the bottom of the tube.
7. The DNA pellet was washed twice with 75% ethanol and left to air dry.
8. 100μL of TE buffer was added to dissolve DNA into stock solution and stored at -20 °C for use.
9. DNA quality was checked by 0.8% agarose gel electrophoresis.

### SRAP analysis

361 SRAP primer pairs, produced by combining 19 forward primers with 19 reverse primers were used in screening for polymorphic primers. Out of the 361 primer pairs, 29 were selected as polymorphic primers without too many thin bands.

These 29 SRAP primer pairs were used to screen all the 473 accessions for polymorphisms. The PCR reactions to analyse the SRAP markers consisted of template DNA (20 ng·μL^-1^)1.0 μL, 10×Buffer 2.0 μL, Mg^2+^ (25 mmoL·L^-1^) 2.5 μL, Taq Enzyme (4 U·μL^-1^) 0.4 μL, dNTPs (10 mmoL·L^-1^) 0.2 μL, SRAP Primers (2 μmoL·L^-1^) 1.0 μL+ 1.0 μL, and 11.9 μL ddH_2_O in a total reaction volume of 20 μL.

Amplification was performed in a PTC-20 Thermal Cycler following the process: pre-denaturation at 94°C for 5 min→ denaturation at 94°C for 1 min→ annealing at 35°C for 1 min→ extension at 72°C for 1 min, 5 cycles→ denaturation at 94°C for 1 min→ annealing at 50°C for 1 min→ extension at 72°C for 1min, 30–35 cycles→72°C extension for 7min→stop reaction at 4°C.

A 6% non-denaturing polyacrylamide gel each piece consisting of 14 ml TBE (Tris; boric acid; EDTA disodium salt), 14 ml 30% acrylamide (29 acrylamide: 1 bisacrylamide), 0.5 ml 10% (w/v) ammonium persulfate and 20 μL TEMED, was prepared to segregate the PCR products. After gel electrophoresis, the gel was silver stained to aid in visualizing of polymorphic bands.

### Statistical and data analysis

Every SRAP primer combination was given a score (‘1’ for the presence or ‘0’ for the absence of bands in each accession), and a binary matrix was generated. SPSS 18.0 was used for cluster analysis to obtain clusters of 473 wild castor materials from South China.

Genetic diversity parameters, the proportion of polymorphic loci (P%), average number of alleles per locus (A_p_), average number of effective alleles per locus (*A*_*e*_), mean expected heterozygosity per locus (*H*_*e*_) were computed for each primer combination using POPGENE 1.32 [32].

Scored data was converted to frequency data and frequency-based distance calculated from Nei’s genetic distance [33] was estimated using POWERMARKER [34]. A phylogenetic tree was constructed for the three populations, using unweighted pair group method average UPGMA from the clustering of the OTUs with POWERMARKER [34] to analyse the genetic diversity and kingship among the three locations.

Genetic differentiation between samples was assessed using Nei’s gene diversity index (GST) in POPGENE; gene flow was estimated from GST, expressed as (Nm) = 0.5 (1- GST)/GST [35, 36, 37].

Population structure was analyzed using the program STRUCTURE [38], and the results were input into STRUCTURE HARVESTER [39] to determine the best K (number of clusters) value. We conducted 10 runs for each value of K from 1–20 with both length of burning period and number of MCMC reps after burning set at 10000, an admixture model for ancestry and allele frequencies set as independent.

## Results and analysis

### DNA extraction

The extracted DNA was subjected to agarose gel electrophoresis to check the quality. The DNA bands were clear, bright and there was no tailing phenomenon. The extracted DNA was nearly transparent, flocculent and elastic indicating that the improved CTAB method could obtain higher quality and higher DNA concentrations to be used for PCR amplification.

### SRAP amplification

**Fig 2:**
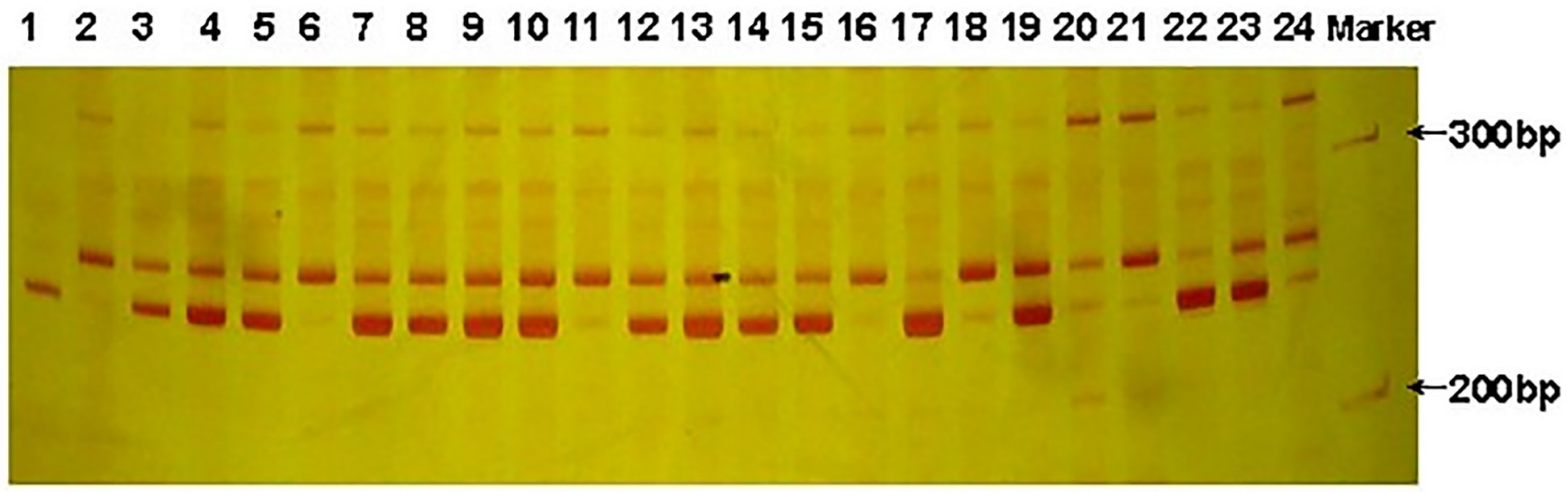
Amplified profiles of different materials by SRAP primers M2E7.

473 wild castor accessions from South China were amplified by 29 pairs of SRAP polymorphic primers and a total of 203 clear, bright and stable bands were amplified, including 169 polymorphic bands, with an 83.25% polymorphism. The number of bands amplified by each pair of primer combination was between 2 and 8, among which primers M1E9, M6E4, and M17E3 amplified the most bands with 8. Primer M19E1 had the lowest, with only 2 bands. The amplified DNA bands ranged from 100 to 500bp, mostly between 200-400bp. Average alleles per locus (A_p_) was 1.801 while the effective alleles per locus (*A*_*e*_) was 1.713. The percentage of polymorphic loci (P) ranged from 50–100% with an average of 90.04% (Table 2). Fig 2 shows the amplified band pattern of the SRAP primer M2E7, with high band definition, few bands, and obvious polymorphism.

**Table 2:** Genetic diversity of each pair of SRAP markers.

Marker	A_p_	*A*_*e*_	*H*_*e*_	P(%)	Marker	A_p_	*A*_*e*_	*H*_*e*_	P(%)
M1E3	1.778	1.5184	0.3148	88.89	M9E1	2	1.91	0.4762	100
M1E9	1.867	1.8736	0.4623	93.33	M9E8	1.333	1.6474	0.3929	66.67
M2E2	2	1.7644	0.4293	100	M10E16	1.333	1.501	0.3337	66.67
M2E4	2	1.9031	0.4733	100	M15E5	2	1.2678	0.2102	100
M2E5	2	1.6954	0.4099	100	M15E12	2	1.9489	0.4868	100
M2E6	2	1.8184	0.4467	100	M17E2	1.833	1.7822	0.4357	91.67
M2E7	2	1.655	0.3796	100	M17E3	1.667	1.8974	0.4719	83.33
M2E13	1	1.0555	0.0516	50	M10E17	1.778	1.5248	0.3119	88.89
M3E7	2	1.8824	0.4688	100	M17E9	1.733	1.5744	0.329	86.67
M3E9	2	1.8964	0.472	100	M17E11	1.556	1.7586	0.4309	77.78
M4E1	1.667	1.7526	0.4238	83.33	M17E12	1.733	1.3287	0.2206	86.67
M5E7	1.833	1.9111	0.4759	91.67	M18E10	2	1.9096	0.4756	100
M6E4	2	1.8718	0.4649	100	M18E16	2	1.9206	0.4793	100
M6E5	1.333	1.555	0.3532	66.67	M19E1	1.778	1.8965	0.4696	88.89
M6E6	2	1.67	0.3882	100					
**Average**	**1.801**	**1.713**	**0.398**	**90.04**					

A_p_: average number of alleles per locus; *A*_*e*_: average number of effective alleles per locus; *H*_*e*_: Mean expected Heterozygosity per locus; P: percentage of polymorphic loci.

### Cluster analysis

**Fig 3:**
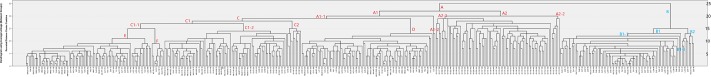
A dendrogram of 473 wild castor materials based on SRAP analysis.

The genetic distance of 473 wild castor materials ranged from 1.04 to 25.02 with an average genetic distance of 13.03. In the dendrogram, the 473 wild castor materials were grouped into two major groups at a distance of 25.02 (Fig 3). However, more clustering groups existed between 5.21 and 13.32. Group A mainly consisted of materials from Guangxi and Hainan, but also materials from West Guangdong. Group B consisted of other 93 materials from West Guangdong, which were classified as one set at the genetic distance of 15.09.

111 and 10 Guangxi materials were clustered into subgroups A2 and C2 at the genetic distance of 20.01 and 14.86 respectively. At the genetic distance of 15.09 and 11.79, 175 West Guangdong materials clustered into group B (93) and subgroup E (82) respectively; and 48 Hainan materials clustered separately into subgroup F at the genetic distance of 9.98 (Fig 3). Not all materials from the same region could be clustered into the same group, therefore, the genetic grouping of materials was not entirely affected by the region. Nonetheless, an obvious trend existed that the groups were closely related to regions. This showed that there was an evident genetic differentiation between regions and there were immigrants in each region, which seemed to be artificially carried to the collection area; confirming what we were told by some local residents that some materials were brought into collection area for medicinal use from other places where their relatives lived.

### Determination of genetic diversity index

The subpopulations, West Guangdong and Guangxi had the same percentage of polymorphic loci (P) and average number of allele per locus (A_p_). However, average number of effective allele per locus (*A*_*e*_) and mean expected heterozygosity per locus (*H*_*e*_) of West Guangdong and Guangxi were higher than Hainan, and those of West Guangdong were slightly higher than Guangxi (Table 3). The results showed that all indicators in Hainan were the lowest.

**Table 3:** Genetic diversity index of subpopulations.

Sub Populations	A_p_	*A*_*e*_	*H*_*e*_	P(%)
West Guangdong	1.977	1.6955	0.3869	97.7
Guangxi	1.977	1.6347	0.3591	97.7
Hainan	1.7701	1.4476	0.2627	77.01

A_p_: average number of allele per locus; *A*_*e*_: average number of effective allele per locus; He: Mean expected heterozygosity per locus; P: percentage of polymorphic loci.

### Phylogenetic tree construction

**Fig 4:**
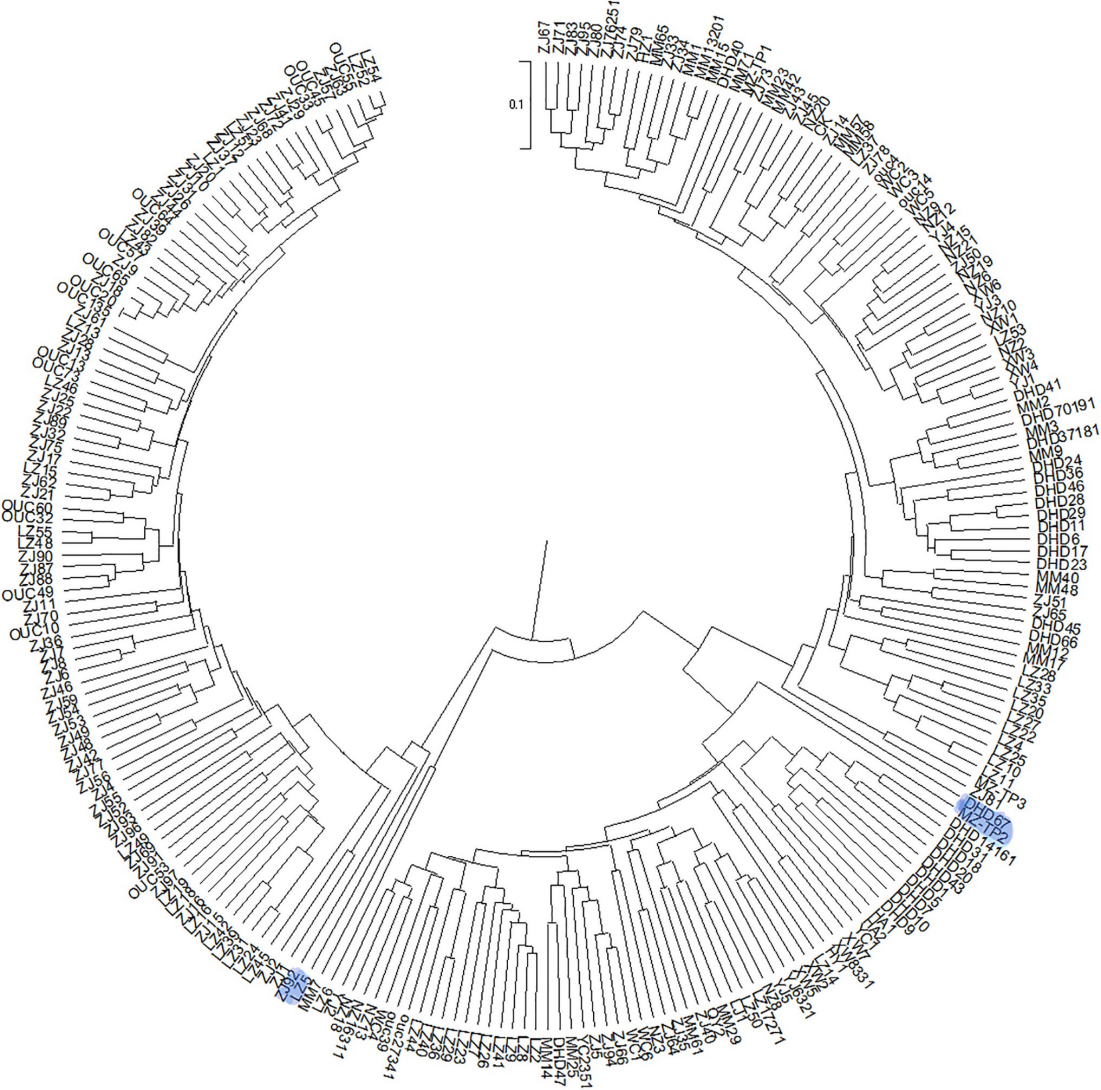
Phylogenetic tree of 245 castor materials from West Guangdong (GD). LZ5, ZJ92, DHD67, MZ-TP2 have been highlighted for easy identification.

**Fig 5:**
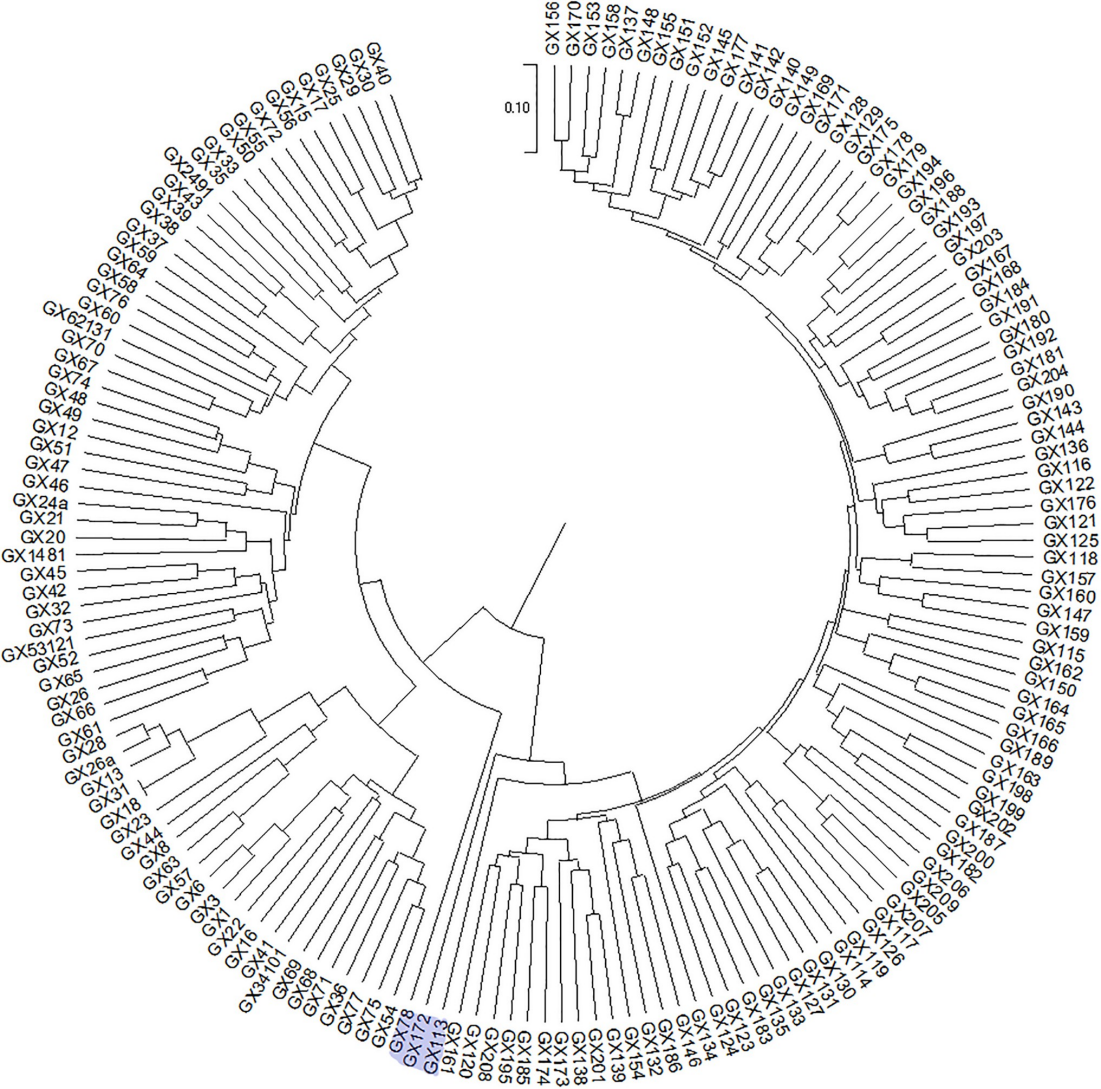
Phylogenetic tree of 168 castor materials from Guangxi (GX). GX172, GX 113, GX78 have been highlighted for easy identification.

**Fig 6:**
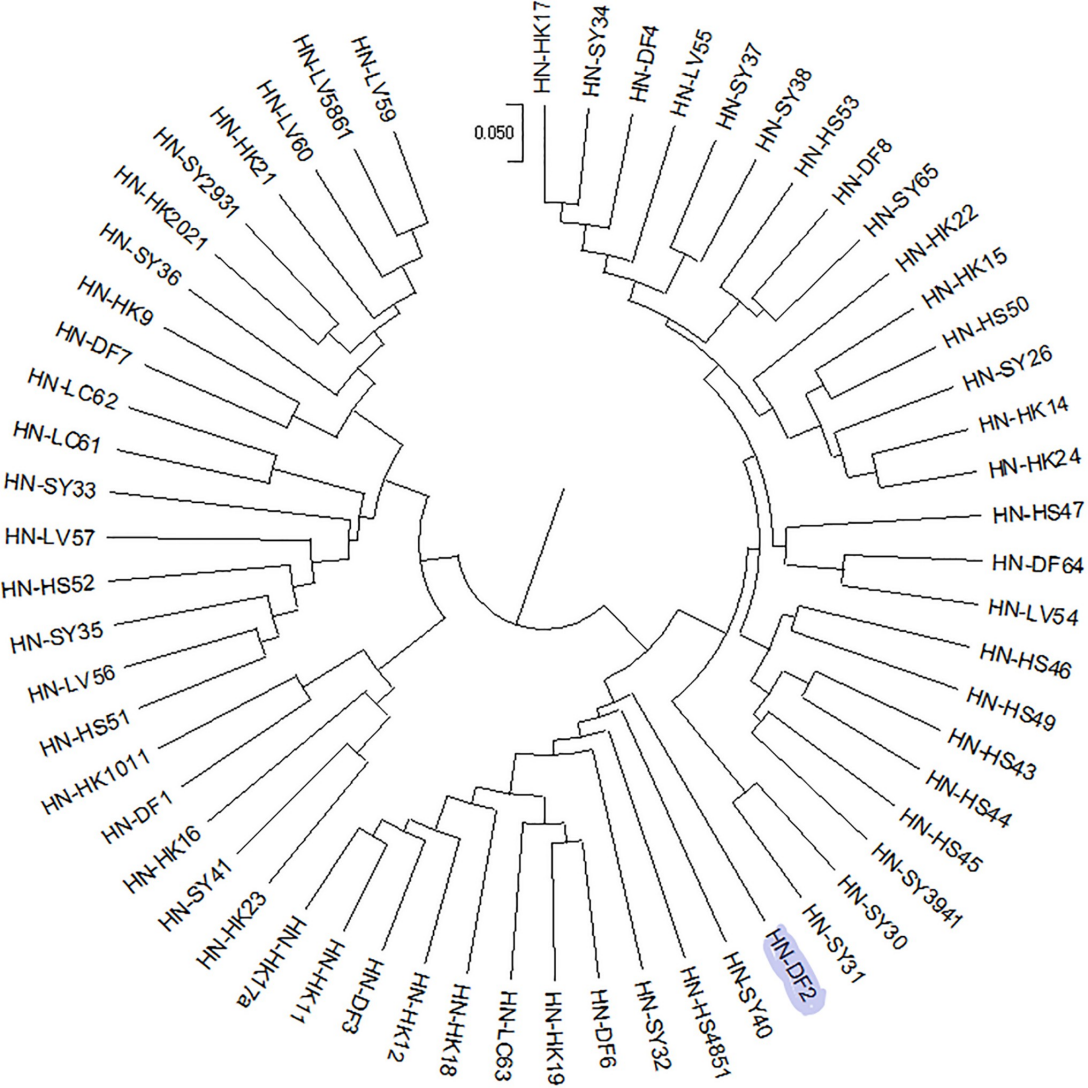
Phylogenetic tree of 60 materials from Hainan (HN). HNDF-2 has been highlighted for easy identification.

Due to the excessive number of materials in this experiment, large amount of data, and the complexity of the system, no phylogenetic tree containing all materials was made. However, phylogenetic trees of the regions were constructed individually to make a better assessment of their diversity. After screening, 245 from West Guangdong (GD), 168 materials from Guangxi (GX) and 60 from Hainan were used to construct phylogenetic trees (Figs 4–6).

The phylogenetic trees showed that the region with the accessions having the highest genetic distance was Guangxi, followed by West Guangdong. The highest genetic distance that the accessions from Hainan had was 0.23 which was lower as compared to the highest accessions in Guangxi (0.35) and West Guangdong (0.28). This showed that the materials in Guangxi differentiated more greatly. GX172 was the root of 96 accessions out of the 168 total accessions in Guangxi indicating 57.14% which is a sign of high ancestry. GX113, the immediate descendant of GX 172 also branched into 95 descendants. 70 progenies branching from GX78 also confirmed 41.67% (Fig 5). LZ5, DHD67 and MZ-TP2 from West Guangdong branched into 94, 87 and 60 progenies out of a total of 245 accessions respectively (Fig 4). HNDF-2 branched into 11 progenies out of the 60 accessions (Fig 6). From this, we inferred that the wild castor materials in South China were evolved from accessions such as GX172, GX 113, GX78, LZ5, ZJ92, DHD67, MZ-TP2 and HNDF-2.

### Population structure

**Fig 7:**
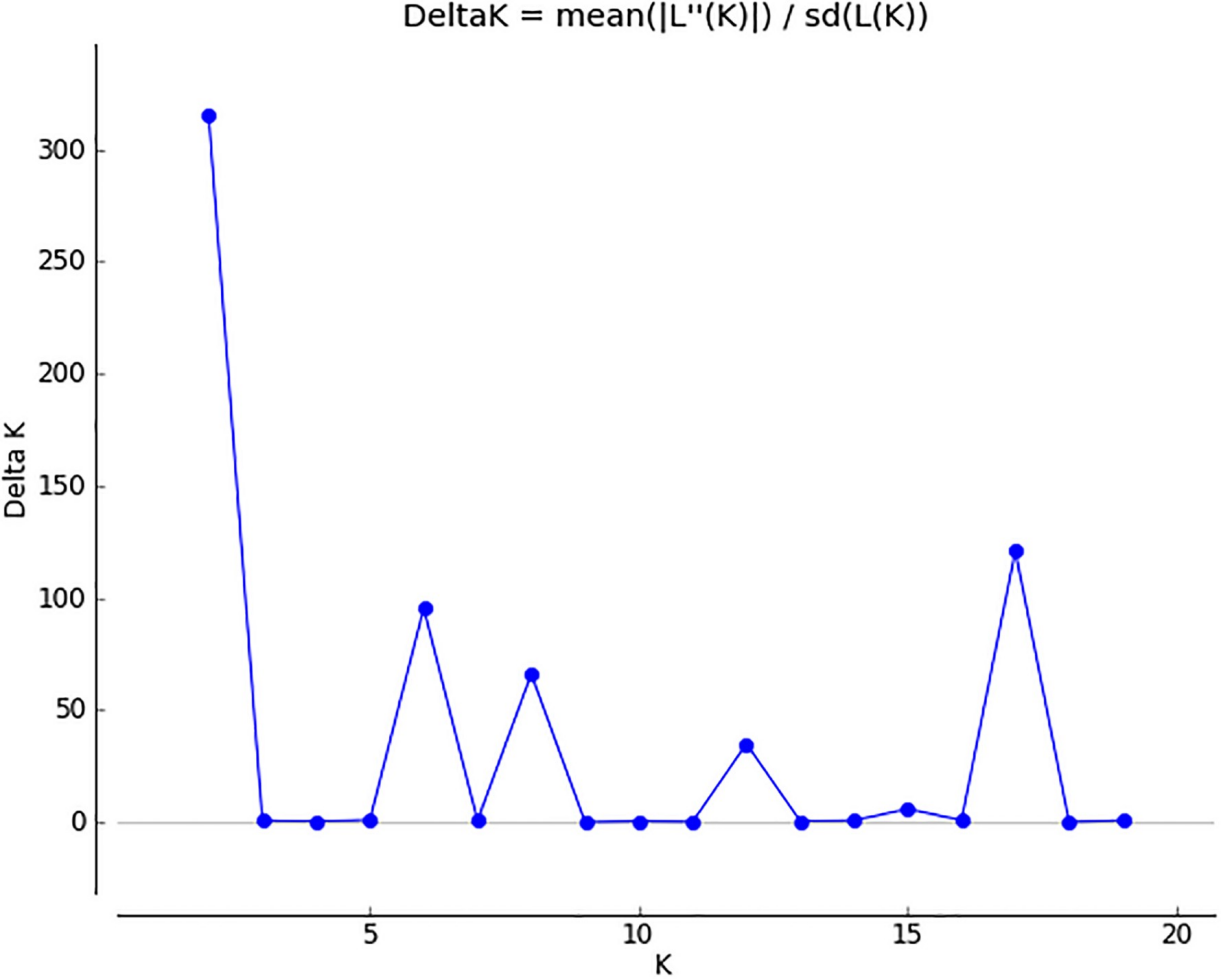
Estimates of the rate of the slope of the log probability curve (DK) plotted against K.

**Fig 8:**
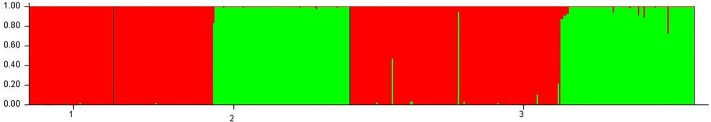
Assignment of 473 castor accessions into two clusters (red and green) with STRUCTURE 2.3.4.

The results output from STRUCTURE HARVESTER showed a clear maximum ΔK at K = 2 (Fig 7) where all the populations were divided into two (2) clusters (Fig 8). The average distance (expected heterozygosity) between the individuals in clusters 1 and 2 was 0.3372 and 0.3342 respectively. The division of the accessions into two clusters was consistent with the UPGMA (Fig 3) which divided the accessions into two major groups.

### Genetic differentiation and geneflow

POPGENE was used to analyze the genetic differentiation between the populations, with a GST value of 0.1075(<0.5), a moderate level of differentiation was observed. The Geneflow estimate result, Nm value, was 4.1543 (>4), showing an extensive geneflow existed between regions.

## Discussion and conclusion

### The usefulness of SRAP markers

With a polymorphic percentage of 83.25%, an average percentage of polymorphic loci of 90.04%, an average number of allele per locus (A_p_) of 1.801 and average number of effective allele per locus (*A*_*e*_) of 1.713, the SRAP molecular marker showed its usefulness and effectiveness in identifying the genetic diversity of wild castor germplasm resources.

The results showed high reproducibility of the markers. The technique is simple, the information is abundant, and the amplified bands are numerous, clear and easy to distinguish.

Moreover, any forward primer can be combined with any reverse primer pairwise, so a large number of primers can be assembled using only a small number of primers to improve the efficiency of primers. Therefore, it has been widely applied to the gene/QTL mapping, genetic map construction, genetic diversity analysis, etc. in different crops. This confirmed the work by many researchers on SRAP markers and their various functions [29, 40, 41]

### The genetic diversity of wild castor in South China

Firstly, the genetic diversity indices and clustering results showed that the genetic diversity of wild castor materials in South China was poor and low, consistent with the previous reports [17, 19, 20, 23] that recorded a minimum to medium genetic diversity in castor using different kinds of molecular markers. The materials in this experiment were clustered in groups mainly at a genetic distance range of 4.67–5.84, indicating that the wild materials in South China were rich in genetic polymorphism at a low molecular level.

Secondly, although not all the materials from the same region could be clustered in the same group, the clustering was closely related to the regions. Most materials from the same region did enter the same group or subgroup; for example, 93 (37.96%) West Guangdong materials in group B, 82 (33.47%) West Guangdong materials in subgroup E, 111 (66.07%) Guangxi materials in subgroup A2, and 48 (80%) Hainan materials in subgroup F. This conclusion was different from the previous reports [17–19, 23] which showed a lack of geographically structured genetic population.

Thirdly, the genetic homogeneity and differentiation existed within and between regions. The genetic homogeneity resulted from the bottleneck effect of a population where the materials sharing ancestry in the same area were related closely. Geographic segregation easily exists, and hybridisation rate is low. The genetic homogeneity between regions may be due to the immigrants from each other and subsequent randomly mating within population. The genetic differentiation within a region mainly arose from gene mutation and crossing recombination especially crossing with immigration. The genetic differentiation between regions was mainly because of the founder effect of each population.

Fourthly, the genetic diversity of the three regions followed the descending order from West Guangdong, Guangxi, to Hainan. The subpopulation West Guangdong had the highest average number of effective allele per locus (*A*_*e*_) and mean expected heterozygosity per locus (*H*_*e*_), mainly because West Guangdong, is relatively flat and located between Guangxi and Hainan. There is frequent gene exchange as compared to the mountainous area Guangxi and completely isolated island Hainan. With the same average number of allele per locus (A_P_) and percentage of polymorphic loci (P), there was not much difference between materials from West Guangdong and Guangxi. However West Guangdong was slightly higher than Guangxi. The largest genetic differentiation existed in Guangxi subpopulation. With a much larger territory and diverse ambient conditions and much-reserved nature due to the presence of many mountains, more variation was produced compared to West Guangdong and Hainan. The genetic diversity and differentiation of Hainan subpopulation were the lowest. This was probably because Hainan is an isolated island, separated from mainland China with lower contamination of foreign genes, low heterogeneity between materials and the differentiation of genetic diversity mainly caused by gene mutation and recombination. However, Hainan’s unique climate has caused its phenotype to be more diverse. Therefore, we finally determined the genetic diversity as West Guangdong > Guangxi > Hainan. GST is the relative measure of genetic differentiation between samples, and it is an indication as to whether the greater variation is within or between the samples of interest [42]. When GST<0.5, it means the majority of variation exists within samples [43, 44]. In our experiment, GST was 0.1074 which indicated that the variation mainly existed within the populations, consistent to the clustering results. If Nm > 4, then local populations belong to one panmictic (randomly mating) population [45]. The *Nm* value of our experiment (Nm = 4.1543) could describe the low heterozygosity of wild castor materials in South China.

However, this experiment did not cover the whole of South China entirely as the materials from Guangdong were only from the West. In this regard, they cannot be generalised as a representative of all of South China castor.

## Supporting information

S1 Table Binary data of SRAP markers. (XLSX)

S2 Table List of accessions from West Guangdong, Guangxi and Hainan. (DOCX)

S3 Table Frequency based genetic distances. Frequency Based Genetic Distances.
(XLSX)
