# Supplementary material for: SRAP analysis of the genetic diversity of wild castor (Ricinus communis L.) in South China
Source: PLoS One. 2019 Jul 11;14(7):e0219667. doi: 10.1371/journal.pone.0219667 (PMC6622549; doi:10.1371/journal.pone.0219667)
Supplement: S2 Table — (DOCX) [file pone.0219667.s002.docx]

| **List of accessions from West Guangdong** | | | | | | | | | |
| --- | --- | --- | --- | --- | --- | --- | --- | --- | --- |
| Accessions | | | | | | | | | |
| ZJ1 | ZJ31 | ZJ59 | ZJ83 | LZ22 | LZ54 | DHD47 | WC6 | YJ1 | MM23 |
| ZJ2 | ZJ32 | ZJ61 | ZJ84 | LZ23 | LZ55 | DHD66 | XW1 | YJ2 | MM25 |
| ZJ4 | ZJ33 | ZJ62 | ZJ87 | LZ24 | DHD1 | DHD67 | XW2 | YJ3 | MM29 |
| ZJ5 | ZJ34 | ZJ63 | ZJ88 | LZ25 | DHD5 | DHD70191 | XW3 | YJ4 | MM31 |
| ZJ6 | ZJ35 | ZJ64 | ZJ89 | LZ26 | DHD6 | OUC10 | XW4 | YJ5 | MM32 |
| ZJ7 | ZJ36 | ZJ65 | ZJ90 | LZ27 | DHD7 | OUC13 | XW5 | YJ6321 | MM33 |
| ZJ8 | ZJ37 | ZJ66 | ZJ91 | LZ28 | DHD9 | OUC20 | XW6 | HA2 | MM34 |
| ZJ9 | ZJ39 | ZJ67 | ZJ92 | LZ29281 | DHD10 | OUC32 | XW7 | HA1 | MM35 |
| ZJ10 | ZJ40 | ZJ68 | ZJ93 | LZ30 | DHD11 | OUC33 | XW8331 | QY1 | MM36 |
| ZJ11 | ZJ41 | ZJ69 | ZJ94 | LZ31 | DHD14161 | OUC45 | NZ2 | QY2 | MM37 |
| ZJ12 | ZJ42 | ZJ70 | ZJ95 | LZ31 | DHD17 | OUC49 | NZ3 | HZ1 | MM38 |
| ZJ13 | ZJ43 | ZJ71 | ZJ96 | LZ33 | DHD18 | OUC53 | NZ6 | HY1 | MM40 |
| ZJ14 | ZJ44 | ZJ72 | ZJ97 | LZ35 | DHD20 | OUC55 | NZ8 | MM1 | MM41221 |
| ZJ15 | ZJ45 | ZJ73 | LZ1 | LZ36 | DHD23 | OUC57 | NZ9 | MM2 | MM42 |
| ZJ16 | ZJ46 | ZJ74 | LZ2 | LZ37 | DHD24 | OUC60 | NZ12 | MM3 | MM48 |
| ZJ17 | ZJ48 | ZJ75 | LZ3261 | LZ38 | DHD28 | OUC64 | NZ13 | MM6 | MM57 |
| ZJ18 | ZJ49 | ZJ76251 | LZ4 | LZ39 | DHD29 | OUC65 | NZ15 | MM7 | MM58 |
| ZJ20 | ZJ50 | ZJ78 | LZ5 | LZ42 | DHD31 | OUC73 | NZ16311 | MM9 | MM61 |
| ZJ21 | ZJ51 | ZJ79 | LZ13 | LZ43 | DHD36 | OUC135 | NZ18 | MM10 | MM65 |
| ZJ22 | ZJ52 | ZJ77 | LZ15 | LZ45 | DHD37181 | LJ1 | NZ20 | MM12 | MM71 |
| ZJ23 | ZJ53 | ZJ78 | LZ16 | LZ46 | DHD40 | WC1 | MZ-TP1 | MM13201 |  |
| ZJ25 | ZJ54 | ZJ79 | LZ18 | LZ48 | DHD41 | WC2 | MZ-TP2 | MM14 |  |
| ZJ26 | ZJ55 | ZJ80 | LZ19 | LZ49 | DHD43 | WC3 | MZ-TP3 | MM15 |  |
| ZJ28 | ZJ56 | ZJ81 | LZ20 | LZ51 | DHD45 | WC4 | YC1 | MM17 |  |
| ZJ29 | ZJ57 | ZJ82 | LZ21 | LZ52 | DHD46 | WC5 | YC2351 | MM18 |  |

ZJ, LZ, DHD, NZ, MM, and all others in table above belong to West Guangdong

Note: Area - 44,585.25 sq km

Altitude of West Guangdong is 38m (125ft) above sea level,

| **List of accessions from Guangxi** | | | | | | | | | |
| --- | --- | --- | --- | --- | --- | --- | --- | --- | --- |
| Accession | | | | | | | | | |
| GX1 | GX2491C | GX41 | GX58 | GX75 | GX126 | GX143 | GX160 | GX177 | GX194 |
| GX3 | GX25 | GX42 | GX59 | GX76 | GX127 | GX144 | GX161 | GX178 | GX195 |
| GX6 | GX261 | GX43 | GX60 | GX77 | GX128 | GX145 | GX162 | GX179 | GX196 |
| GX8 | GX262 | GX44 | GX61 | GX78 | GX129 | GX146 | GX163 | GX180 | GX198 |
| GX12 | GX28 | GX45 | GX62131 | GX113 | GX130 | GX147 | GX164 | GX181 | GX199 |
| GX13 | GX29 | GX46 | GX63 | GX114 | GX131 | GX148 | GX165 | GX182 | GX200 |
| GX1481 | GX30 | GX47 | GX64 | GX115 | GX132 | GX149 | GX166 | GX183 | GX201 |
| GX15 | GX31 | GX48 | GX65 | GX116 | GX133 | GX150 | GX167 | GX184 | GX202 |
| GX16 | GX32 | GX49 | GX66 | GX117 | GX134 | GX151 | GX168 | GX185 | GX203 |
| GX17 | GX33 | GX50 | GX67 | GX118 | GX135 | GX152 | GX169 | GX187 | GX204 |
| GX18 | GX34101 | GX51 | GX68 | GX119 | GX136 | GX153 | GX170 | GX188 | GX205 |
| GX20 | GX35 | GX52 | GX69 | GX120 | GX137 | GX154 | GX171 | GX189 | GX206 |
| GX21 | GX36 | GX53121 | GX70 | GX121 | GX138 | GX155 | GX172 | GX190 | GX207 |
| GX22 | GX37 | GX54 | GX71 | GX122 | GX139 | GX156 | GX173 | GX186 | GX208 |
| GX23 | GX38 | GX55 | GX72 | GX123 | GX140 | GX157 | GX174 | GX191 | GX209 |
| GX2491A | GX39 | GX56 | GX73 | GX124 | GX141 | GX158 | GX175 | GX192 |  |
| GX2491B | GX40 | GX57 | GX74 | GX125 | GX142 | GX159 | GX176 | GX193 |  |

Gx- Guangxi series

Note: Area - 235,001 sq km

Altitude of Guangxi is 2,141 m (7,024ft)

| **Table 3. List of accessions from Hainan** | | | | | | | | | |
| --- | --- | --- | --- | --- | --- | --- | --- | --- | --- |
| Accession | | | | | | | | | |
| HN-DF1 | HN-DF8 | HN-HK15 | HN-HK2021 | HN-SY2931 | HN-SY35 | HN-SY41 | HN-HS4851 | HN-LV54 | HN-LV60 |
| HN-DF2 | HN-HK9 | HN-HK16 | HN-HK21 | HN-SY30 | HN-SY36 | HN-HS43 | HN-HS49 | HN-LV55 | HN-LC61 |
| HN-DF3 | HN-HK1011 | HN-HK17 | HN-HK22 | HN-SY31 | HN-SY37 | HN-HS44 | HN-HS50 | HN-LV56 | HN-LC62 |
| HN-DF4 | HN-HK11 | HN-HK17 | HN-HK23 | HN-SY32 | HN-SY38 | HN-HS45 | HN-HS51 | HN-LV57 | HN-LC63 |
| HN-DF6 | HN-HK12 | HN-HK18 | HN-HK24 | HN-SY33 | HN-SY3941 | HN-HS46 | HN-HS52 | HN-LV5861 | HN-DF64 |
| HN-DF7 | HN-HK14 | HN-HK19 | HN-SY26 | HN-SY34 | HN-SY40 | HN-HS47 | HN-HS53 | HN-LV59 | HN-SY65 |

HN-Hainan series

Note: Area- 60,438 sq km

Altitude of Hainan is 1,867m (6,125ft).
